# A cluster of Legionnaires' disease in Belgium linked to a cooling tower, August–September 2016: practical approach and challenges

**DOI:** 10.1017/S0950268819001821

**Published:** 2019-12-20

**Authors:** N. Hammami, V. Laisnez, I. Wybo, D. Uvijn, C. Broucke, A. Van Damme, L. Van Zandweghe, W. Bultynck, W. Temmerman, L. Van De Ginste, T. Moens, E. Robesyn

**Affiliations:** 1 Agency for Care and Health, Infection Prevention and Control, Flemish Community, Ghent, Belgium; 2 Department of Microbiology and Infection Control, National Reference Centre for Legionella Pneumophila, Universitair Ziekenhuis Brussel, Vrije Universiteit Brussel, Brussels, Belgium; 3 Agency for Care and Health, Environmental Health, Flemish Community, Ghent, Belgium; 4 Pneumology Department, Sint-Blasius Hospital, Dendermonde, Belgium; 5 European Centre for Disease Prevention and Control, Surveillance and Response Support Unit, Stockholm, Sweden; 6 Department of Global Public Health, Karolinska Institutet, Stockholm, Sweden

**Keywords:** Community outbreaks, geographical information systems, Legionnaires’ disease

## Abstract

A cluster of Legionnaires' disease (LD) with 10 confirmed, three probable and four possible cases occurred in August and September 2016 in Dendermonde, Belgium. The incidence in the district was 7 cases/100 000 population, exceeding the maximum annual incidence in the previous 5 years of 1.5/100 000. Epidemiological, environmental and geographical investigations identified a cooling tower (CT) as the most likely source. The case risk around the tower decreased with increasing distance and was highest within 5 km. *Legionella pneumophila* serogroup 1, ST48, was identified in a human respiratory sample but could not be matched with the environmental results. Public health authorities imposed measures to control the contamination of the CT and organised follow-up sampling. We identified obstacles encountered during the cluster investigation and formulated recommendations for improved LD cluster management, including faster coordination of teams through the outbreak control team, improved communication about clinical and environmental sample analysis, more detailed documentation of potential exposures obtained through the case questionnaire and earlier use of a geographical information tool to compare potential sources and for hypothesis generation.

## Introduction

Legionnaires' disease (LD) is pneumonia caused by *Legionella* spp. and is often severe [1]. Early diagnosis and appropriate treatment are important to improve the clinical outcome. Infection occurs by inhalation of aerosolised contaminated water particles. Aerosol-generating devices such as cooling towers (CTs), spa-pools, showers and fountains can infect people indoor or outdoor, and can cause outbreaks [2–7].

LD is a notifiable infection in Belgium to allow for source identification and adequate control measures to prevent new infections [1, 8, 9]. In 2015, Belgium reported 165 cases of LD to the European Centre of Disease Prevention and Control (ECDC) [10], a national notification rate of 1.47/100 000.

In this paper, we describe a cluster of LD in Dendermonde, a district in Belgium, with a population of 198 494 [11]. On 9th September 2016, a third case of LD within 2 weeks was notified to the public health authorities. This led to case investigations and active case finding. A total of 10 confirmed, three probable and four possible LD cases with onset of disease between 20th August and 12th September 2016 were detected. We used the ECDC outbreak investigation toolkit and the geographical information tool for identification of potential sources [12, 13]. We also formulated recommendations for future outbreak investigations.

## Methods

### Case definitions

Case finding was done by contacting hospitals and primary care physicians. Cases had to have onset of symptoms from 1st August 2016 onwards and had to live or work in Dendermonde district in the 14 days before onset of symptoms. The clinical and laboratory criteria to define and classify cases were in line with the EU surveillance definitions [14].

### Epidemiological investigations

The epidemiological investigations were performed by the Infection Control team of the Agency for Care and Health. The following clinical data were collected from each suspect case: date of symptom onset and diagnosis, type and result of diagnostic test, underlying disease or risk factors and recent admission to hospital. Each case was interviewed over phone, using a standardised questionnaire consistent with the ECDC trawling questionnaire [12], to collect information about residence, profession and workplace, stays away from home (including history of travel or hospitalisation) and possible exposure to aerosol-generating devices during 14 days prior to symptom onset. Data entry was done in Excel 2010^®^. The most likely exposure period under the scenario of a point source contamination was estimated by subtracting the minimum and maximum incubation periods from respectively the first and last dates of onset [15].

### Microbiological investigations

A commercial urine antigen test (Alere BinaxNow^®^) was used to detect the *Legionella pneumophila* serogroup 1 soluble antigen in all suspect cases. Blood and respiratory samples were sent to the National Reference Centre (NRC) for *Legionella* in Brussels for confirmation of diagnosis. Traditional culturing techniques on buffered charcoal yeast extract agar (BCYE) and *Legionella* BMPA Selective Agar (Oxoid, UK), as well as real-time polymerase chain reaction (PCR) based on *mip*-gene were applied on respiratory samples [16, 17]. Identification of isolates was performed by matrix-assisted laser desorption-ionisation time of flight mass spectrometry using a Microflex LT mass spectrometer with MALDI Biotyper 3.1 software and Bruker Reference Library v5.0.0.0 (5989 MSP) (Bruker Daltonik GmbH, Bremen, Germany). Serogroup typing (differentiation between serogroups 1 and 2–15) was performed by latex agglutination using a Microgen *Legionella* latex kit (Microgen Bioproducts Ltd., UK). The NRC performs sequence-based typing on clinical samples with *L. pneumophila* serogroup 1 isolates for discrimination of strains [18, 19].

For the serological examination, an indirect immunofluorescence test was used to detect *L. pneumophila* pooled serogroup 1–6 (*L. pneumophila* 1–6 IFA, Meridian, Villa Cortese, Italy). The NRC does not distinguish serogroup 1 from the other serogroups via serology. When the urine antigen test was negative, a second serological test was performed after 6 weeks to detect a seroconversion or fourfold increase in the antibody level.

### Environmental investigations

In Belgium, the registration of CTs is mandatory (only) in Flanders and regional legislation requires the existence of a legionella management plan based on a risk assessment, with at least bi-annual sampling [20]. Possible sources of infections were identified via the case questionnaires, by screening existing aerial photos for aerosol-producing infrastructure, and during a field visit in the affected area. Samples were taken by the Flemish Environmental Agency in accordance with international protocols and sent to the laboratory for culture and serogroup identification (differentiation between serogroups 1 and 2–14). They were analysed with appropriate methods (WAC/V/A/005, based on ISO 11731, ISO 11731-2 and NEN 6265): diluted and non-diluted and heat pre-treated samples were plated on GVPC extract (glycine, vancomycin, polymyxin and cycloheximide) and BCYE [21]. The *Legionella* strains isolated after 7 days were sent to the NRC for matching with available human samples. Sample results and *Legionella* management plans were assessed during on-site visits by the Environmental Health team. No monoclonal subtyping was performed on clinical or environmental isolates.

### Use of outbreak investigation tools

Post-outbreak, we used the ECDC geographical information tool [13] to map cases' residences and we visualised case density in Dendermonde district.

Potential sources were also mapped in the tool, and the attack rate per 100 000 population was estimated in concentric zones around each potential source by dividing the case count by the population in each concentric zone [13]. Relative risks were estimated with the risk outside the largest concentric zone but within the two bordering provinces as baseline.

For the evaluation of the cluster investigation, we assessed retrospectively the six aspects listed in the ECDC LD outbreak investigation toolkit [12]: (1) *summarising background information*; (2) *data collection* and convening of a coordinating outbreak control team; (3) *data management* and (4) *data analysis* to generate a hypothesis of a common outbreak source; (5) support to public health decisions about *control measures* and (6) appropriate *communication* throughout the investigation. These aspects are also reflected in the Flemish LD outbreak investigation procedure.

## Results

### Epidemiological investigations

Upon the alert, clinicians in one hospital mentioned an increase in admissions for pneumonia since 25th August. They identified – mostly retrospectively – 23 suspect patients admitted in two hospitals, with date of onset between 20th August and 12th September. The case definition for patients possibly belonging to the community cluster was set on 12th September. Among the 17 retained (confirmed, probable or possible) cases, one was on immunosuppressive treatment, one suffered from chronic pulmonary disease, one reported to be a smoker and four were over 65 years old. Overall, the mean age was 55 years (range 35–77 years). The male to female ratio was 4.8. Four cases (24%) were admitted to an intensive care unit and none died.

**Fig. 1.**
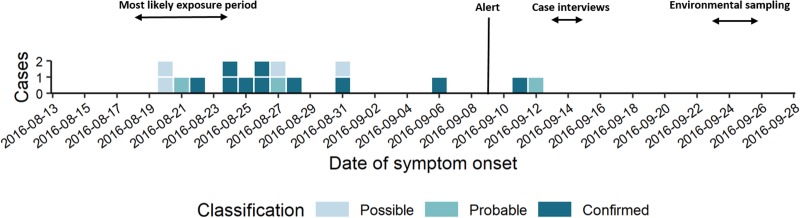
Epi curve by date of symptom onset (*n*  =  17).

The short epi curve (Fig. 1) [22] and proximity of case residences suggested a common source with a short contaminated aerosol release most likely between 18th and 24th August.

### Microbiological investigations

On 15th September, one respiratory sample and 16 serum samples were sent to the NRC with a follow-up serum sample after 6 weeks for 15 cases. Of the 23 initial suspect patients, 10 were classified as confirmed LD cases (four with positive urine antigen and six with significant rise in the antibody level for serogroups 1–6 in paired samples), three as probable cases (single high antibody titre serogroups 1–6), four as possible cases (pneumonia in a cluster setting but with negative baseline serology without follow-up sample), while six were discarded because they did not meet the case definition (e.g. absent seroconversion or fourfold rise in a paired sample). *L. pneumophila* serogroup 1 was cultured in the single available respiratory sample and was identified as ST48.

### Environmental investigations and control measures

Based on the questionnaire replies, no common indoor or outdoor exposure was identified, except for two cases who used the same carwash. Seven cases lived or worked within 2 km of the industrial area in Dendermonde and two of those were neighbours. During the 14 days before onset of symptoms, one case had travelled abroad (7 days in France), but was not part of a travel-associated cluster.

Due to the clustering in time and space and the lack of a common place visited, we hypothesised that the cases could be caused by a contaminated CT. Six CTs were listed based on the register and map inspection. CT6 was not functioning during the incubation period and was excluded as a potential source. All identified CTs were involved in industrial processes and had *Legionella* management plans. Samples from the five CTs were taken on 23rd September. The samples from CT1, CT2 and CT5 were negative. CT3 and CT4 had positive cultures with, respectively 500 and 2600 colony forming units/litre (cfu/l) of *L. pneumophila* serogroups 2–14. A second sample from CT3, taken on the same day by the company, resulted in 40 000 cfu/l. CT3 had also a water sample result from July 2016 of 1850 cfu/l with *L. pneumophila* serogroups 2–14 after which CT3 had been emptied and cleaned and activities had resumed without control sample. Based on the result of 23rd September, the *Legionella* management plan was adjusted to include biocide shock treatment every 2 months. The consecutive samples remained <400 cfu/l. As the isolate found in the human sample belonged to serogroup 1 and isolates from the CTs to serogroups 2–14, no sequence-based typing was performed on the environmental samples. All five CTs were assessed on-site and no major deficiencies were detected in the *Legionella* management plans and no specific control measures were imposed, except for CT3.

Further, the car wash used by two cases, and a truck wash in the abovementioned industrial area identified during the field visit, were potential sources because of possible aerosol production. Samples from the truck wash were taken on 26th September and were negative. Sampling at the car wash was performed in February 2017 and samples were positive up to 590 000 cfu/l of *L. pneumophila* serogroup 1. The car wash had initially not been considered a plausible common source for the cluster and a communication problem with the owner had further delayed the sampling. This car wash sample was not available for sequence-based typing. An inspection visit to the car wash detected deficiencies in the production and storage of warm water at 35–40 °C. Recommendations were made to sustainably decrease the risk of *Legionella* growth.

### Use of outbreak investigation tools

#### Summarising epidemiological background information

The first case was notified on 28th August and an alert was triggered by the provincial infection control team when a third confirmed case was notified on 9th September in the same district within an interval of 2 weeks. In the previous 5 years, only between zero and three cases had been notified per year in the Dendermonde district (annual incidence 0–1.5/100 000).

#### Data collection, management and analysis to generate a hypothesis of a common source and support decision about measures

During evaluation of the cluster, the ECDC geographical information tool was used to generate hypotheses about the most likely source.

**Fig. 2.**
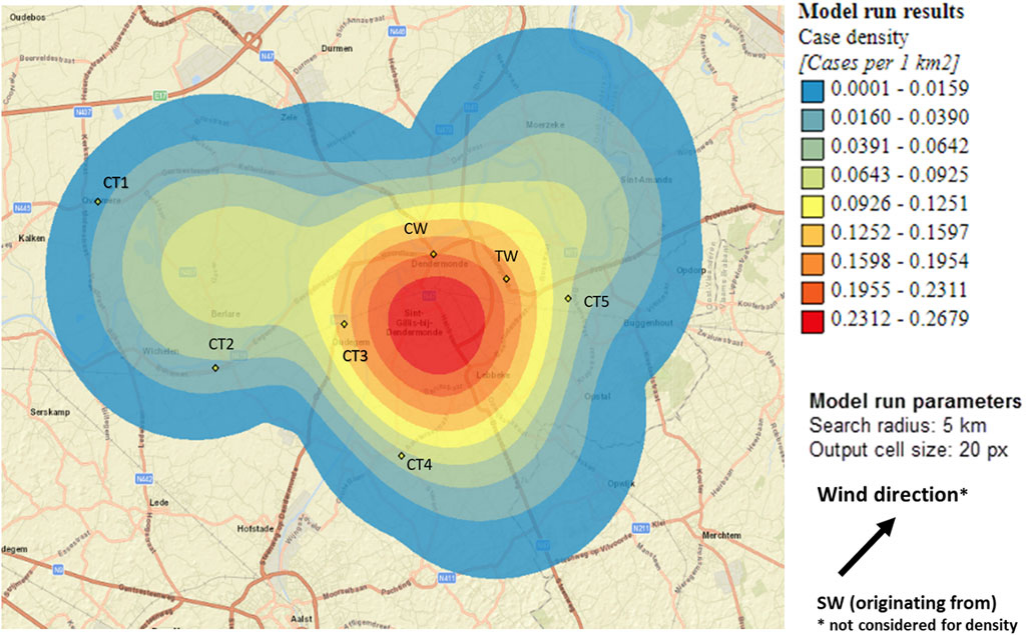
Spatial density of confirmed and probable case residence locations (*n*  =  13) and seven potential source locations CT, cooling tower; CW, car wash; TW, truck wash.

Sixty-nine percent of confirmed and probable cases lived in the municipality Dendermonde (20 cases/100 000) and the highest incidence (55 cases/100 000 inhabitants) was observed in the submunicipality Sint-Gillis-Dendermonde (Fig. 2). Cases were up to 8 km away from the epicentre of the cluster and the maximum distance between any two cases was 12 km.

The disease incidence in the entire district was 7 cases/100 000 population, but the observed attack rate varied considerably in relation to different potential sources. We choose to look at different distances, depending on the type and height of the potential source, with distances up to 10 km for CTs and up to 5 km for the car wash and truck wash. Post-outbreak mapping showed the highest risk estimates were around the car wash, the truck wash, CT3 and CT5 (Table 1). Predominant wind direction during the outbreak period was south-west [23].

**Table 1.** Attack rates per 100 000 and relative risks in concentric zones with increasing distance (km) from each potential source

Distance	<2.5 km		2.5–5 km		5–7.5 km		7.5–10 km		>10 km but in district	
Source	AR	RR	AR	RR	AR	RR	AR	RR	AR	RR
CT1	0.0	0.0	3.9	8.2	0.0	0.0	0.6	1.3	0.5	Ref.
CT2	0.0	0.0	3.8	37.7	1.9	18.7	1.6	15.5	0.1	Ref.
CT3	5.6	NA	6.5	NA	1.8	NA	0.4	NA	0.0	Ref.
CT4	0.0	NA	5.9	NA	0.4	NA	1.8	NA	0.0	Ref.
CT5	2.9	29.5	7.0	71.8	0.8	8.0	0.5	5.3	0.1	Ref.
	<2 km		2–3 km		3–4 km		4–5 km		>5 km but in district	
Source	AR	RR	AR	RR	AR	RR	AR	RR	AR	RR
CW	8.8	69.9	6.2	49.5	9.9	78.3	2.3	18.2	0.1	Ref.
TW	4.2	33.2	11.2	88.5	8.1	64.1	0.0	0.0	0.1	Ref.

**Fig. 3.**
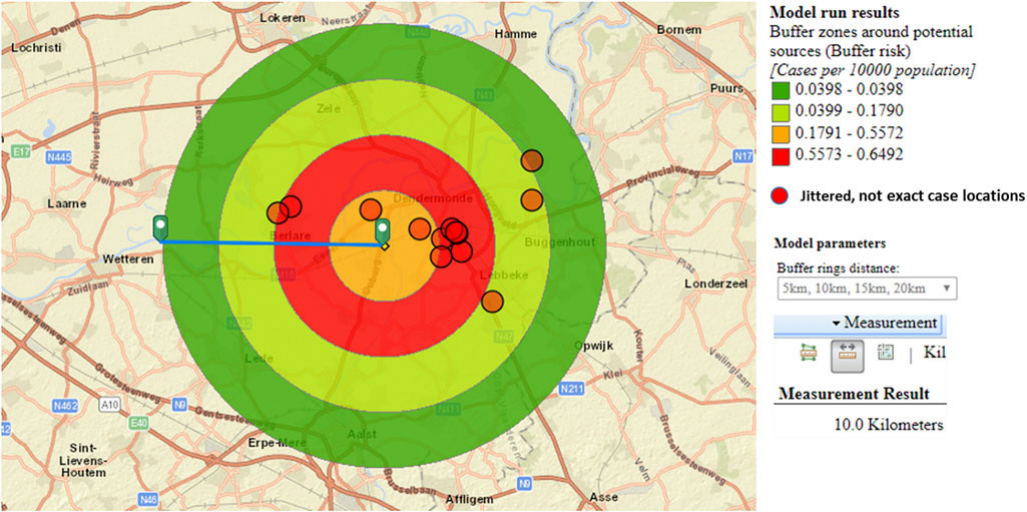
Case risk within concentric zones with increasing distance from CT3 (10-km radius).

We hypothesised that the attack rate decreases with increasing distance from the source, as illustrated by Nygard *et al*. [24]. This is the case for CT3 (Fig. 3). CT5 showed a similar attack rate pattern as CT3, but microbiological results were negative, the highest risk was in the opposite direction of the expected based on the predominant wind direction of the area, and cases lived up to 12 km away. For CT1 most cases were 10 km away from the tower, and CT2 and CT4 had attack rates equal to zero in the 2.5 km around the tower, making these sources less likely.

For the car wash, the attack rate in the immediate neighbourhood of less than 2 km was highest of all sources, but the evolution of the attack rate over distance (based on the location of the residences of cases) did not decrease and peaked at 3–4 km.

#### Communication and stakeholder evaluation

On 23rd September, other hospitals in the province and general practitioners were alerted. The mayors of involved municipalities were also informed on 23th September and were debriefed on 21st November. The communication service of the public health authorities prepared for potential press releases.

On 16th December, all involved stakeholders gathered to review the management of the cluster. Overall, the outbreak investigation effectively consisted of all aspects described in the Flemish procedure for LD outbreak management and the ECDC toolkit upon which it was built [12]. Nevertheless, points for improvement were identified. There was a delay between the date of onset and the date of confirmation of many cases: median 30 days (range 2–75 days). This was partly due to the use of paired serology diagnostics. Environmental samples were taken 2 weeks after cluster notification. This delay was due to several reasons, e.g. the absence of an initial hypothesis about a likely source, the late convening of the outbreak control team, on 9th October, staffing difficulties and lack of business continuity planning, and delays in relation to diagnostics and case classification. We further identified challenges with communication: clear arrangements between involved teams had been hampered by the late convening of the outbreak control team, and environmental samples had not been treated as a priority. Finally, the overall risk communication by the public health teams with involved clinical doctors and mayors was found to have been fragmented.

## Discussion

### Summarising background information

We described a cluster of LD in a Belgian district with 10 confirmed, three probable and four possible cases with onset of disease between 20th August and 12th September 2016. This was considerably more than the maximum of three LD cases per year notified in this district during 2011–2015.

This was the second most important cluster of LD in Belgium. The first occurred in 1999 among visitors to a fair and involved 41 confirmed, 14 probable and 39 possible cases [2]. This outbreak, caused by exposition of a contaminated spa pool, resulted in a higher number of cases, quicker case reporting and case confirmation, and the identification of a common place visited by all cases, the fair, leading to faster implementation of control measures.

The median time from date of onset to notification in the Dendermonde cluster is not exceptionally long, compared to those reported across the European Union/European Economic Area (EU/EEA) Member States with a median of 17 days (range 10–42 days) for 2015 for travel-associated cases [10]. However, the delay in confirming the total number of cases deferred common understanding of the cluster situation, leading to a postponement in environmental investigations.

### Data collection, management and analysis to generate the hypothesis of a common source and support decision about measures

A case-control study was considered to identify common outdoor exposure [3, 25]. It was not done due to low number of confirmed cases, lack of resources and difficulty in defining time-varying spatial exposure and study controls.

Based on outbreak reporting in the EU [10] we know that most outbreaks are of small size and in the majority of community outbreaks the source is not identified. The use of the ECDC geographic information tool allowed identifying a most likely source of infection. Whereas the attack rate was highest around the car wash, the truck wash, CT3 and CT5, all attack rates were lower (maximum 11/100 000 population) than in large and explosive outbreaks. In Sarpsborg, Norway, attack rates up to 270/100 000 population were reported with sources further apart from each other, possibly easing the analysis of varying risks over space [24]. The proven *Legionella* positivity, before and during the cluster, in samples of CT3, in combination with the plausible evolution of case risk around the tower, suggested CT3 as the most likely source. CTs are known to be high risk installations and can cause outbreaks at a distance [26, 27]. Around CT3, the attack rate decreased with increasing distance from the tower, with most cases living north-east of the tower which coincided with predominant south-west wind direction. Adequate and sustained measures were taken by the owner of the CT.

In the 4 months prior detection of high levels of *L. pneumophila* serogroup 1 in the carwash, no possibly related cases were reported, while no control measures had been taken. For the car wash to have been the source, cases must have come nearer to it than their respective residences. Information on case movements in the 14 days before falling ill was not collected in sufficient detail to assess the proximity to this potential source. This underscores the need for future outbreak data tools to not only capture point data such as residences, work or leisure places, but also allow the collection of transient linear spatial attributes, such as transport routes [28].

As in many reported outbreaks [29, 30], no match was found between patient and environmental samples from the suspected source. The presence of *L. pneumophila* from another serogroup in a sample from two CTs confirmed that conditions for growth of this micro-organism were present. We cannot exclude false negative samples, e.g. due to delay in sampling of environmental sources and a lower concentration of *Legionella* at the time of sampling than at the time of transmission [29]. False negative results can further occur because standard culture plates used in environmental samples are easily overgrown by other bacteria [31]. It is also possible that the actual source in our study was not identified and was therefore not sampled. Between 2000 and 2010, only 2.3% of the isolates (2/86) in the Belgian database were ST48 [17] and this was the first isolation of ST48 since 2008. The source of these isolates has never been determined, suggesting that these strains may be present in particular environmental niches unexplored so far [31–35]. The importance of these niches may be underestimated, as they may not yet be considered in source investigations and fall outside the legislative framework for (high) risk installations.

### Limitations

A limitation of our investigation is the use of serology for the confirmation of six additional cases. The EU case definition includes a significant rise in the specific antibody level to *L. pneumophila* serogroup 1 in paired serum samples. In Belgium, the NRC only uses a grouped identification of groups 1–6. The urine antigen test was used in all patients, but has also limitations as it fails to detect other serogroups of *L. pneumophila* than serogroup 1 or other species of *Legionella*. The specificity for the BinaxNOW^®^ is high (99%) but the sensitivity is 75% and dependent of the severity of disease and use of antibiotics [36]. Furthermore, *Legionella* antigen in urine samples can be detected 1–3 days after the onset of the disease but can last for 1 year [36]. Other techniques, such as culture or PCR followed by sequence-based-typing of lower respiratory samples are more reliable to confirm a case, but could only be applied on one respiratory sample in our cluster investigation. No respiratory samples were obtained from haemodynamically stable patients, due to the absence of productive cough, the perceived need for an invasive procedure as broncho-alveolar lavage to obtain an appropriate sample, and prompt initiation of antibiotic treatment. There is a large variation between EU Member States in the percentage retrieval of respiratory samples, and lessons can be learned from countries such as Denmark where culture confirmations accounted for 41% of diagnoses [10].

Another limitation is regarding the use of the geographic information tool. The tool was, rather than during the ongoing investigation, only retrospectively used to its full extent, allowing post-hoc to gather more evidence to identify CT3 as the most likely source [37]. The tool, while easy in use, is limited in its flexibility to assess attack rates at different distances for different kinds of sources, e.g. for comparing the effect of far reaching aerosol plumes from CTs *vs.* the assumed shorter distance aerosol from a car wash. Furthermore, the attack rates are estimated in concentric zones around identified potential sources and do not take into account other relevant factors (e.g. wind direction and velocity). Therefore, the output of the tool requires careful interpretation by the investigators. Nevertheless, we showed that the use of freely available tools can support the difficult source identification task.

### Recommendations

To improve epidemiological data collection, more attention should be given to document detailed itineraries of cases during the 14 days before onset of symptoms, and the outbreak investigation toolkit should be used early on in the investigation. To improve the gathering of microbiological evidence and facilitate a better reporting of positive and negative results, a table including all cases of a cluster with relevant details should be readily available to the NRC. To increase availability of human respiratory samples, detailed instructions on how to collect those were integrated in the guidelines. To prioritise and better prepare the environmental investigations, the outbreak control team should convene early on, from the first cluster signal. To prioritise the analysis and follow-up of environmental samples linked with a cluster *vs.* single cases, a two track system was initiated for the lab. All these recommendations were integrated in a revised procedure, including also a flow chart visualising the parallel actions needed for epidemiological and environmental investigations, the identification of back-up staff for each member of the outbreak control team and improved coordination and communication flows between involved teams.

We believe that the approach and recommendations from this investigation are relevant in the light of the many clusters and outbreaks across the EU/EEA that continue to be reported without identified source [10]. We showed that the use of freely available tools can support the difficult source identification task during a LD outbreak.
